# Anthropometric characteristics and long-term trends among olympic male water polo players from former Yugoslavia

**DOI:** 10.3389/fpubh.2026.1797720

**Published:** 2026-03-09

**Authors:** Jovan Gardasevic

**Affiliations:** Faculty for Sport and Physical Education, University of Montenegro, Niksic, Montenegro

**Keywords:** anthropometry, BMI, body height, body weight, former Yugoslavia, Olympic Games, water polo

## Abstract

Anthropometric characteristics are recognized as important determinants of elite performance in water polo. Although secular trends in body height, body weight, and BMI have been documented in various sports, long-term historical analyses of Olympic water polo players from a single, highly successful sporting system are lacking. This retrospective population-based study analyzed anthropometric data of male water polo players representing former Yugoslavia at the Olympic Games between 1956 and 1988. Body height, body weight, and body mass index (BMI) were examined using descriptive statistics and trend analyses to identify long-term morphological changes across Olympic cycles. A pronounced secular increase in body height was observed across Olympic cycles, accompanied by a moderate increase in body weight. In contrast, BMI values remained relatively stable over time, indicating proportional morphological development rather than excessive body weight gain. Considering these trends and the profiles of the former Yugoslav national teams that achieved the greatest successes at the Olympic Games, it is evident that similar anthropometric characteristics should be taken into account when selecting top level players for elite squads.

## Introduction

It is well known that success in sport depends on many different factors, such as motor, physiological, anthropometric, technical, tactical, and psychological components. Anthropometric characteristics are one of these factors influencing sporting success across a wide range of disciplines, particularly in sports that require considerable strength, endurance, and direct physical contact with opponents (1, 2). Body height, body weight, and body mass index (BMI) are closely associated with the biomechanical and physiological demands of sport and frequently serve as important criteria in the athlete selection process at the elite level (3).

Secular trends in anthropometric characteristics, defined as long-term changes in body size and composition across generations, have been well documented both in the general population and in sport (4, 5). Previous research indicates a progressive increase in body height and body weight among athletes throughout the 20^th^ century, which has been attributed to improvements in living conditions, nutrition, and training methodologies (6).

Water polo is recognized as one of the most physically and energetically demanding team sports. It requires an optimal combination of body height, body weight, muscular strength, anaerobic and aerobic endurance, as well as a high level of technical and tactical proficiency (7–9). Unlike many other team sports, water polo is performed in an aquatic environment, which further emphasizes the importance of body morphology, buoyancy, and the strength-to-body-weight ratio (10). Previous studies have shown that successful water polo players are, on average, taller and heavier than athletes in other team sports, while maintaining relatively stable BMI values that reflect a high proportion of lean muscle weight (9, 11).

Numerous studies indicate that anthropometric characteristics, including body height and body weight, are important determinants of selection and performance in water polo, especially at the elite level (12, 17). Greater body height provides a competitive advantage in duels, blocking, and shooting actions, while higher body weight, when combined with an optimal body mass index, contributes to stability, strength, and effectiveness during physical contact with opponents (9).

The Socialist Federal Republic of Yugoslavia, a former large state located in the Balkan Peninsula in Southern Europe, represented a unique and rare example of long-term dominance in international water polo. The men's national water polo team of former Yugoslavia won a total of three Olympic gold medals (Mexico City 1968; Los Angeles 1984; Seoul 1988) and four silver medals (Helsinki 1952; Melbourne 1956; Tokyo 1964; Moscow 1980), ranking it among the most successful national teams in the history of the sport.

Such continuity of success, despite substantial changes in the political, social, and institutional context, suggests the presence of long-term stable factors related to the sporting system, training methodology, selection criteria, and the anthropometric profile of players (1, 6). In this regard, former Yugoslavia and its successor states represent a unique “natural experiment” for examining long-term anthropometric patterns in elite sport.

Despite extensive research on the anthropometric characteristics of contemporary water polo players (10, 11), there remains a notable lack of historical, longitudinal analyses focusing on Olympic level athletes originating from a single, consistently successful sporting system across multiple decades. In particular, no previous study has systematically examined secular trends in body height, body weight, and BMI among Olympic water polo players from former Yugoslavia, a country and sporting culture characterized by prolonged dominance and structural continuity even after political dissolution. Addressing this gap may provide valuable insights into the role of stable selection criteria and long-term athlete development processes in elite sport performance.

The aim of this study was to examine the anthropometric characteristics and long-term trends in body height, body weight, and body mass index (BMI) among male Olympic water polo players from former Yugoslavia. Based on relevant literature documenting pronounced secular trends in body height and body weight in the general population during the 20^th^ century (1, 6), as well as findings indicating that elite athletes largely reflect these trends, it will be reasonable to assume that Olympic water polo players will follow general population patterns. However, the scientific contribution of this study will not lie in a possible confirmation of the existence of secular trends. Rather, it will lie in a systematic analysis of their dynamics, magnitude, and specific manifestations within a clearly defined population of elite water polo players over an extended period. This study will provide a more precise assessment of whether changes in body height, body weight, and body mass index (BMI) among Olympic water polo players will be proportional to, accelerated relative to, or structurally modified compared with general population trends. It will also enable an evaluation of whether long-term selection and training processes will result in sport specific morphological adaptations. In this way, the study will contribute to a deeper understanding of the long-term effects of selection and sport-specific demands on the morphological profile of elite water polo players.

The analysis of historical data of this kind is of particular importance, as it enables the examination of morphological development in elite athletes across extended time periods, an approach that remains underrepresented in contemporary literature. Understanding historical anthropometric patterns will provide essential context for the interpretation of current findings and may contribute to projections of future trends in elite water polo.

## Methods

### Study design

This study employed a retrospective, descriptive analytical design based on a secondary analysis of historical data on male water polo players who represented the former Socialist Federal Republic of Yugoslavia at the Olympic Games during the second half of the 20^th^ century. Data from nine consecutive Summer Olympic cycles, spanning from 1956 to 1988, were analyzed, covering all Olympic appearances of this national team up to its final participation. Although the study spans more than three decades, it does not represent a true longitudinal design, as different athletes participated in each Olympic cycle. Accordingly, the findings should be interpreted as repeated cross-sectional cohort comparisons rather than longitudinal tracking of individual morphological development. The retrospective approach enabled the examination of long-term trends and potential secular changes in fundamental anthropometric characteristics of elite water polo players within a specific historical and sporting context.

### Participants

The study sample comprised a total of 95 male water polo players who competed for the Olympic national team of former Yugoslavia between 1956 and 1988. Participants took part in nine consecutive Olympic Games: Melbourne 1956 (*n* = 6), Rome 1960 (*n* = 8), Tokyo 1964 (*n* = 11), Mexico City 1968 (*n* = 11), Munich 1972 (*n* = 11), Montreal 1976 (*n* = 11), Moscow 1980 (*n* = 11), Los Angeles 1984 (*n* = 13), and Seoul 1988 (*n* = 13). The Seoul 1988 Olympic Games represented the final Olympic appearance of the Yugoslav national water polo team prior to the dissolution of the state in 1991. Although complete and consistent data on basic anthropometric characteristics were available for all participants, allowing their inclusion in the statistical analyses, certain Olympic cycles included relatively small sample sizes (e.g., *n* = 6 and *n* = 8). These smaller cohorts may reduce statistical stability and increase susceptibility to individual variability influencing mean values. Accordingly, results for specific Olympic cycles should be interpreted with consideration of potential sampling variability.

### Variables

In this study, basic anthropometric variables relevant for assessing the morphological profile of elite water polo players were analyzed. These included body height, expressed in centimeters (cm); body weight, expressed in kilograms (kg); and body mass index (BMI), calculated as the ratio of body weight to the square of body height (kg/m^2^). The selected variables represent standard and widely used indicators in sports science and anthropological research, enabling comparisons with both earlier and contemporary studies involving similar athletic populations. However, the study relied on secondary data collected across multiple Olympic cycles. Detailed information on measurement procedures, equipment calibration, and testing conditions was not consistently available. Therefore, minor methodological differences across decades may have influenced the recorded values, and full comparability of measurements over time cannot be fully ensured.

### Statistical analysis

Data processing and statistical analyses were performed using IBM SPSS Statistics, version 20.0 (IBM Corp., Armonk, NY, USA). Descriptive statistical methods were applied, including the calculation of means and measures of variability. In addition, analyses of secular trends were conducted to examine changes in anthropometric characteristics across the observed time period. This analytical approach enabled the identification of potential long-term patterns, as well as the assessment of stability or change in the morphological profile of Olympic water polo players from former Yugoslavia across successive Olympic cycles.

## Results

An analysis of basic descriptive statistical parameters (Table 1) is presented for the anthropometric variables: body height, body weight, and body mass index (BMI).

**Table 1:** Anthropometric characteristics of male Olympic water polo players from former Yugoslavia across Olympic cycles.

**Olympic games**	**N**	Mean ± SD			
		**Age (years)**	**Body height (cm)**	**Body weight (kg)**	**Body weight index (kg/m** ^2^ **)**
Melbourne 1956	6	23.83 ± 2.71	180.67 ± 3.93	85.83 ± 6.52	26.26 ± 1.16
Roma 1960	8	24.00 ± 3.85	186.75 ± 5.78	89.12 ± 6.85	25.53 ± 1.16
Tokyo 1964	11	25.27 ± 3.04	187.45 ± 7.26	92.18 ± 9.46	26.20 ± 1.75
Mexico City 1968	11	25.91 ± 2.88	189.91 ± 6.46	94.09 ± 8.43	26.10 ± 2.17
Munich 1972	11	27.73 ± 2.90	188.45 ± 6.52	90.91 ± 8.68	25.60 ± 2.12
Montreal 1976	11	27.27 ± 3.90	190.09 ± 4.72	93.00 ± 8.67	25.70 ± 1.73
Moskva 1980	11	26.36 ± 3.56	189.09 ± 4.55	91.00 ± 7.01	25.44 ± 1.64
Los Angeles 1984	13	23.38 ± 3.12	192.62 ± 5.98	91.85 ± 6.05	24.77 ± 1.42
Seoul 1988	13	23.38 ± 2.63	195.08 ± 4.75	94.08 ± 9.40	24.71 ± 2.08

Table 1 indicates that water polo players from former Yugoslavia were, on average, youngest during the Olympic Games appearances, in Seoul 1988 (23.38 ± 2.63 years), while the oldest average age was recorded at the Munich 1972 (27.73 ± 2.90 years). As shown in Table 1, the lowest mean body height was observed at the first Olympic Games in Melbourne 1956 (180.67 ± 3.93 cm), whereas the greatest mean body height was recorded at the final Olympic Games in Seoul 1988 (195.08 ± 4.75 cm).

The lowest mean body weight was observed at the Melbourne 1956 (85.83 ± 6.52 kg), while the highest mean body weight was recorded at the Mexico City 1968 (94.09 ± 8.43 kg). The lowest average BMI among water polo players from former Yugoslavia was observed at the final Olympic Games in Seoul 1988 (24.71 ± 2.08 kg/m^2^), whereas the highest average BMI was recorded at the first Olympic Games in Melbourne 1956 (26.26 ± 1.16 kg/m^2^).

### Trends across olympic cycles

The analysis of anthropometric characteristics across Olympic cycles revealed variations in the mean values of body height, body weight, and BMI. A pronounced secular increase in body height was observed from the first analyzed Olympic Games in Melbourne 1956 (180.67 cm) to the last in Seoul 1988, where the average player height reached 195.08 cm. Mean body weight increased from Melbourne 1956 (85.83 kg) to Mexico City 1968 (94.09 kg). Body weight values then fluctuated slightly, reaching the highest value again at the last analyzed Games in Seoul 1988 (94.08 kg). Average BMI values remained relatively stable from Melbourne 1956 (26.26 kg/m^2^) to Mexico City 1968 (26.10 kg/m^2^), followed by a slight decreasing trend until Seoul 1988 (24.71 kg/m^2^).

### Graphical analysis

A graphical analysis of the mean anthropometric values of former Yugoslav water polo players across Olympic cycles revealed clear and consistent long-term trends from the 1956 Melbourne Olympics to the 1988 Seoul Olympics. For each anthropometric variable, a separate line graph was constructed, with the year of the Olympic Games as the independent variable.

### Trend in body height

**Figure 1:**
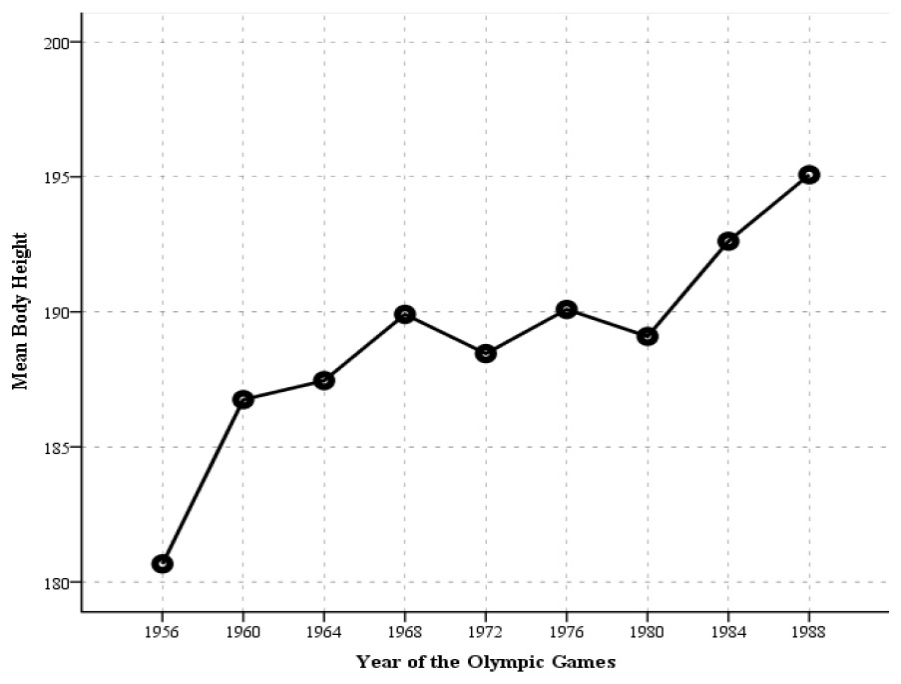
Trend in body height of Olympic male water polo players from former Yugoslavia.

Figure 1 illustrates the secular trend in body height among male water polo players from former Yugoslavia, representing the most pronounced and robust finding of the present study. Mean body height exhibited an almost linear increase across all analyzed Olympic cycles, rising from 180.67 cm at the first analyzed Olympic Games in Melbourne 1956 to 195.08 cm at the final analyzed Olympic Games in Seoul 1988. These findings clearly indicate that increasingly taller players were selected for the former Yugoslav water polo team at the Olympic Games from 1956 to 1988. This pattern may suggest that coaches were likely aware of the importance of player height for success in elite water polo. Taller players have an advantage in aspects of the game involving physical contact, defensive coverage, and spatial dominance.

### Trend in body weight

**Figure 2:**
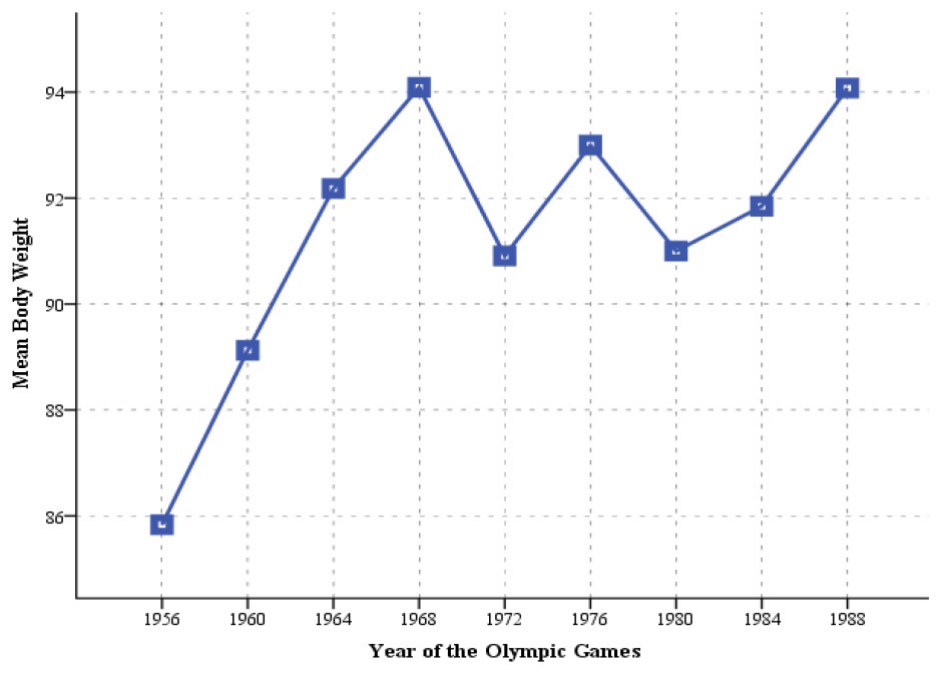
Trend in body weight of Olympic male water polo players from former Yugoslavia.

Figure 2 presents the trend in mean body weight of water polo players across Olympic cycles. The results indicate a consistent increase in body weight from the first analyzed Olympic Games in Melbourne 1956 (85.83 kg) to the Mexico City 1968, at which players from former Yugoslavia recorded the highest mean body weight (94.09 kg). Following the Mexico City 1968, body weight showed some variation; however, by the Seoul 1988 (94.08 kg), mean body weight returned to a level comparable to that observed in 1968. This pattern suggests an adaptation of the athletes' morphological profile from 1968 to the progressively increasing physical demands of the game, without any indication of a disproportionate increase in body mass up to the Seoul 1988.

### Trend in body mass index

**Figure 3:**
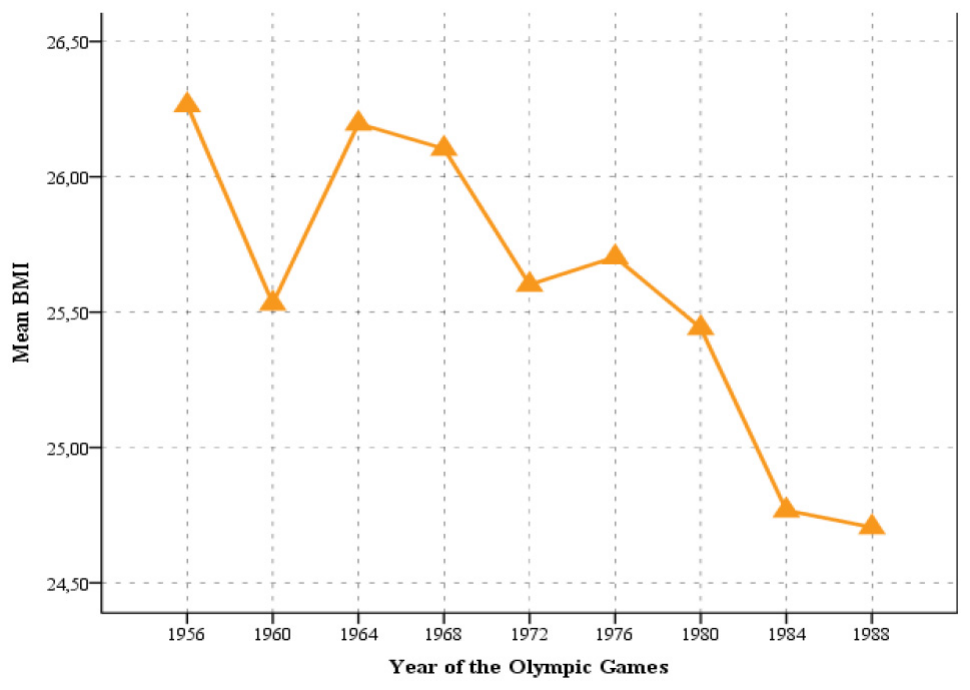
Trend in BMI of Olympic male water polo players from former Yugoslavia.

Figure 3 illustrates modest changes in mean BMI values over the analyzed period. The highest average BMI values were recorded at the first analyzed Olympic Games in Melbourne 1956 (26.26 kg/m^2^). BMI values remained similar until the 1968 Mexico City Olympics (26.10 kg/m^2^). From 1968 to the 1988 Seoul Olympics, a slight but consistent decline in BMI was observed. The lowest average BMI values were recorded at the final analyzed Games in Seoul 1988 (24.71 kg/m^2^). Importantly, the observed secular increase in body height was consistent across nearly all Olympic cycles, suggesting a systematic rather than incidental selection process. In contrast, BMI values remained within a narrow range throughout the analyzed period, indicating that increases in body weight were largely proportional to increases in body height.

## Discussion

The aim of this study was to examine the anthropometric characteristics and long-term trends in body height, body weight, and BMI among male Olympic water polo players from former Yugoslavia. The main findings indicate that the anthropometric profile of these water polo players was characterized by increases in body height and body weight, accompanied by relatively stable BMI values over time.

The results of this study provide a unique historical perspective on long-term anthropometric changes among male water polo players from former Yugoslavia who competed at the Olympic Games between 1956 and 1988. The most prominent finding was a pronounced secular increase in body height, with mean values rising from 180.7 cm at the Melbourne 1956 Olympic Games to 195.1 cm at the Seoul 1988 Olympic Games. Body weight also demonstrated an increasing trend over time, whereas BMI values remained relatively stable, with a slight decreasing tendency observed in the later Olympic cycles. This pattern suggests a long-term association with the selection and development of athletes exhibiting favorable morphological characteristics, potentially reflecting adaptation to the progressively increasing physical demands of elite water polo.

The findings of the present study confirm that Olympic water polo players from former Yugoslavia were, on average, tall athletes, which is consistent with previous research highlighting the importance of body height in water polo performance (9, 11). The pronounced increase in body height observed across Olympic cycles further supports the view that morphological characteristics are an important determinant of success in elite water polo. One of the most frequently cited studies on the anthropometric characteristics of elite water polo players (13) reported a profile of 19 professional athletes with an average body height of 184.5 cm and body weight of 90.7 kg. These results indicate the high physical demands required at the elite level of water polo. These empirical data support the findings of the present study regarding increases in body height and body weight, suggesting that top-level water polo players tend to exhibit greater stature and body weight. Such characteristics were consistently manifested as desirable among the performance-successful water polo selections of former Yugoslavia over time. Furthermore, the study by Lozovina and Pavicić (12) demonstrated significant anthropometric changes among elite water polo players over a 15 year period (1980–1995), including increases in body height and reductions in relative body fat, while total body weight remained stable across generations. These findings are also consistent with the results of the present study. The pronounced increase in body height across Olympic cycles further confirms that this morphological characteristic represents an important factor in elite water polo. Taller players may have a competitive advantage in physical duels, shot blocking, spatial control, and in executing offensive actions near the goal.

The simultaneous increase in body weight, without a corresponding increase in BMI, suggests that changes in body size occurred in a proportional manner, preserving an optimal relationship between body weight and body height. The observed gradual increase in body height across Olympic cycles may reflect broader secular trends in the general population, as well as the effects of progressively refined selection criteria and long-term athlete development within the sporting system (5, 6). While these trends could be influenced by both population-level changes and sport-specific selection mechanisms, the study does not directly compare data with the general population. In this context, it is particularly interesting to examine the relationship between the observed anthropometric trends among Olympic water polo players from former Yugoslavia and the secular trends documented in the general population. Although increases in body height during the second half of the 20^th^ century are well established in the general population, the magnitude and consistency of this increase among elite water polo players suggest a potential deviation from population level patterns. Such deviations may be interpreted as the result of long-term and selective processes in sport, whereby taller individuals are systematically identified, guided, and retained in elite water polo. This raises an important research question as to whether the observed trends are primarily driven by natural secular changes in the population or are further amplified by sport specific selection mechanisms, representing a key implication of this study.

It is particularly important to emphasize that the observed anthropometric trends temporally coincided with the long standing Olympic success of Yugoslav water polo players. Across the nine Olympic Games included in the analysis, former Yugoslavia failed to win a medal on only two occasions, Rome 1960 and Munich 1972. As shown in Table 1, the most favorable morphological profile of water polo players was evident at the final two Olympic Games, Los Angeles 1984 and Seoul 1988, at which former Yugoslavia won two gold medals. These findings support the notion of enhanced player quality in the water, characterized by greater body height and body weight accompanied by stable or slightly lower BMI values.

In a broader context, these results highlight the importance of early identification and targeted development of morphological potential in young athletes, as well as the role of organized sporting systems in shaping optimal physical characteristics through long-term and systematically controlled training processes. Understanding such historical trends may contribute to the improvement of contemporary models of athlete selection and development, not only in water polo but also in other sports with comparable physical and physiological demands.

The body weight of Olympic water polo players from former Yugoslavia was relatively high, which is consistent with the demands of the sport, characterized by frequent physical contact, intense positional battles, and high requirements for strength and stability (10, 17). The increase in body weight observed over time likely reflects changes in training methodologies, particularly the greater emphasis on strength training, whereby increases in body weight predominantly result from gains in muscle mass in response to the escalating physical demands of modern water polo (7). Similar trends have been reported among elite water polo players from other countries, where increases in body weight have occurred alongside the preservation of functional performance capacity (11). This suggests that the development of body composition occurred proportionally, maintaining an optimal balance between body weight and body height. Moreover, the regulation of body weight and preservation of an optimal BMI indicate that the ratio of muscle mass to agility is important in water polo players, accompanied by a reduction in relative body fat (12). This is further supported by the study of López-Laval et al. (14), which demonstrated that trunk length, muscle mass, and biomechanical variables are strongly associated with throwing velocity in elite male water polo players, suggesting that anthropometry is not merely a descriptive characteristic but also a functional component of performance. It is noteworthy that similar trends have been observed in other age groups and sexes. For example, Tan et al. (8) analyzed the anthropometric and fitness characteristics of elite Australian female water polo players and found that taller and heavier athletes performed better in jumping and swimming tests, indicating that body height and body weight remain important factors for functional performance even across different player categories.

One of the notable findings of the present study is the relative stability of BMI values over time, followed by a slight decrease in the later Olympic cycles, despite concurrent increases in body height and body weight. This pattern indicates that increases in body weight occurred proportionally to increases in body height, suggesting the maintenance of an optimal body build and a high proportion of lean muscle mass (2, 3).

These findings further support the notion that, despite its limitations in accurately assessing body composition, particularly in athletic populations with higher levels of muscle mass, BMI may still serve as a useful indicative measure in elite athletes when interpreted within the context of sport-specific demands (15).

Importantly, these long-term anthropometric trends coincide with sustained international success of Yugoslavia and post Yugoslavia national teams, suggesting that systematic selection and long-term development of athletes with favorable morphological characteristics may represent a key structural factor underlying prolonged sporting dominance. These findings support the hypothesis that stable selection criteria and strategically planned long-term athlete development can exert a decisive influence on international success (1, 6). The dissolution of Yugoslavia beginning in 1991 did not mark the end of this sporting continuity. The countries that emerged from the former Yugoslavia, particularly Serbia, Croatia, and Montenegro, maintained and further confirmed a world class level of performance in international sport, most notably in water polo. It can be argued that the dominance of global water polo in the 21^st^ century has largely continued through these successor states. Competing jointly as Serbia and Montenegro, the national team won one Olympic silver medal (Athens 2004) and one bronze medal (Sydney 2000). Following the dissolution of this state union in 2006, Serbia, as an independent nation, won three consecutive Olympic gold medals (Rio de Janeiro 2016; Tokyo 2020; Paris 2024) and two bronze medals (Beijing 2008; London 2012). Croatia secured one Olympic gold medal (London 2012) and three silver medals (Atlanta 1996; Rio de Janeiro 2016; Paris 2024), while Montenegro achieved three fourth place finishes at the Olympic Games (Beijing 2008; London 2012; Rio de Janeiro 2016).

The principal finding of the present study is the existence of a pronounced secular increase in body height among male Olympic water polo players from former Yugoslavia across multiple Olympic cycles. This trend was accompanied by a moderate increase in body weight, while BMI remained relatively stable, indicating proportional development of body composition rather than excessive body weight gain.

A study conducted by Vasiljevic and Gardasevic (16) reported similar anthropometric patterns among elite water polo players from countries that emerged following the dissolution of former Yugoslavia who participated in the Tokyo 2021 Olympic Games (Serbia and Montenegro). Serbian water polo players exhibited mean values of body height of 191.02 cm, body weight of 92.21 kg, and BMI of 25.32 kg/m^2^ (16). In the same study, Montenegrin players demonstrated mean body height of 191.79 cm, mean body weight of 95.95 kg, and BMI of 25.24 kg/m^2^. While mean body height values were slightly lower, body weight, and BMI values were remarkably similar to those observed among water polo players from this region who competed at the Olympic Games during the second half of the 20^th^ century. These findings indicate continuity in the optimal morphological profile required for elite water polo performance among national teams that succeeded former Yugoslavia.

This continuity is particularly noteworthy given the substantial political and institutional changes following the dissolution of former Yugoslavia. The preservation of comparable anthropometric profiles alongside sustained international success among post Yugoslavia national teams suggests that long-term athlete development models, selection criteria, and sport specific performance demands have remained largely consistent over time.

### Strengths and limitations of the study

One of the primary strengths of this study lies in its unique historical scope, encompassing multiple Olympic cycles and enabling the analysis of long-term anthropometric trends among elite male water polo players. The use of data derived from the Olympic Games provides a high level of sample validity, as the Olympics represent the highest level of international competition with clearly defined selection processes. An additional strength is the focus on a single sport discipline, which allows for a more precise interpretation of observed changes without the confounding influence of sport-specific differences across various athletic disciplines.

Furthermore, the results have practical implications for talent identification. The observed patterns of increasing body height, accompanied by proportional maintenance of BMI values, may serve as a reference model for contemporary water polo talent identification programs. In this regard, the study provides an empirical basis for defining the optimal morphological profile of an elite water polo player. This research also suggests that anthropometry may represent an important factor in competitive performance.

Finally, the findings have broader scientific significance as they contribute to the literature on secular trends in elite sport, providing empirical data from a specific geographic and historical context that has previously been underexplored.

However, this study also has several limitations that should be considered when interpreting the findings.

First, the study was based on secondary data obtained from public archives. This implies limited control over measurement methodology and potential differences in measurement procedures between Olympic cycles. Standardization of measurements across decades could not be verified, which may affect the accuracy of comparisons. In addition, incomplete data availability for certain Olympic years and athlete cohorts may have constrained a fully continuous analysis.

Second, the analysis included only basic anthropometric variables (body height, body weight, and BMI), without data on body composition (e.g., body fat percentage, muscle mass), somatotype, or segmental dimensions (e.g., arm length), which have been shown in contemporary research to be significant predictors of performance in water polo.

Third, the sample was limited to a single national team. While this represents a strength in terms of the homogeneity of the selection system, it also limits the generalizability of the findings to other countries and sporting systems with different selection models.

Fourth, the study did not include direct indicators of individual sporting performance (e.g., playing time, goals scored, and team position), meaning that the relationship between anthropometry and performance can only be interpreted indirectly. Moreover, functional abilities and technical-tactical skills were not assessed, which could have provided deeper insight into the complex factors underlying success in water polo.

Finally, the relatively small number of athletes in each Olympic cycle constitutes a methodological limitation inherent to studies of elite athletes, which should be considered when interpreting secular trends.

Nevertheless, despite these limitations, the present findings provide relevant and scientifically grounded insights into long-term changes in the anthropometric profiles of elite water polo players and may serve as a valuable foundation for future research.

## Conclusion

Male Olympic water polo players from former Yugoslavia exhibited an anthropometric profile characterized by a pronounced and nearly linear increase in body height, accompanied by proportional increases in body weight and relatively stable BMI values across successive Olympic cycles. These descriptive trends may be associated with competitive outcomes at the elite level, although the study design does not allow for direct causal inferences.

The observed historical patterns provide a valuable reference for understanding morphological characteristics in elite water polo populations. Considering these trends and the profiles of the former Yugoslav national teams that achieved the greatest successes at the Olympic Games, it is evident that similar anthropometric characteristics should be taken into account when selecting top level players for elite squads.

## Data Availability

The datasets presented in this study can be found in online repositories. The names of the repository/repositories and accession number(s) can be found in the article/Supplementary material.
